# Induction of a Protective Response in Mice by the Dengue Virus NS3 Protein Using DNA Vaccines

**DOI:** 10.1371/journal.pone.0025685

**Published:** 2011-10-21

**Authors:** Simone M. Costa, Anna Paula Yorio, Antônio J. S. Gonçalves, Mariana M. Vidale, Emmerson C. B. Costa, Ronaldo Mohana-Borges, Marcia A. Motta, Marcos S. Freire, Ada M. B. Alves

**Affiliations:** 1 Laboratório de Biotecnologia e Fisiologia de Infecções Virais, Instituto Oswaldo Cruz, Fundação Oswaldo Cruz, Rio de Janeiro, Brasil; 2 Laboratório de Genômica Estrutural, Instituto de Biofísica Carlos Chagas Filho, Universidade Federal do Rio de Janeiro, Rio de Janeiro, Brasil; 3 Laboratório de Tecnologia Virológica, Instituto de Tecnologia em Imunobiológicos, Fundação Oswaldo Cruz Foundation, Rio de Janeiro, Brasil; Fundação Oswaldo Cruz, Brazil

## Abstract

The dengue non-structural 3 (NS3) is a multifunctional protein, containing a serino-protease domain, located at the N-terminal portion, and helicase, NTPase and RTPase domains present in the C-terminal region. This protein is considered the main target for CD4+ and CD8+ T cell responses during dengue infection, which may be involved in protection. However, few studies have been undertaken evaluating the use of this protein as a protective antigen against dengue, as well as other flavivirus. In the present work, we investigate the protective efficacy of DNA vaccines based on the NS3 protein from DENV2. Different recombinant plasmids were constructed, encoding either the full-length NS3 protein or only its functional domains (protease and helicase), fused or not to a signal peptide (t-PA). The recombinant proteins were successfully expressed in transfected BHK-21 cells, and only plasmids encoding the t-PA signal sequence mediated protein secretion. Balb/c mice were immunized with the different DNA vaccines and challenged with a lethal dose of DENV2. Most animals immunized with plasmids encoding the full-length NS3 or the helicase domain survived challenge, regardless of the presence of the t-PA. However, some mice presented clinical signs of infection with high morbidity (hind leg paralysis and hunched posture), mainly in animal groups immunized with the DNA vaccines based on the helicase domain. On the other hand, inoculation with plasmids encoding the protease domain did not induce any protection, since mortality and morbidity rates in these mouse groups were similar to those detected in the control animals. The cellular immune response was analyzed by ELISPOT with a specific-CD8+ T cell NS3 peptide. Results revealed that the DNA vaccines based on the full-length protein induced the production of INF-γ, thus suggesting the involvement of this branch of the immune system in the protection.

## Introduction

Dengue is an important public health problem with high rates of morbidity and mortality in most tropical and subtropical areas of the world. Currently, there are more than 2.5 billion people at risk of infection throughout the world. It is estimated that 50–100 million dengue infections occur annually and about 250–500 thousand patients develop the most severe symptoms of the disease, which can lead to death [1]–[4].

Dengue is a mosquito-borne disease caused by the dengue virus (DENV), a member of the *Flaviviridae* family and *Flavivirus* genus, which consists of four closely related but antigenically distinct serotypes, DENV1-4 [5]. Infection may be asymptomatic or result in a diversity of illnesses, which ranges from acute febrile dengue fever (DF) to severe disease forms, including dengue hemorrhagic fever (DHF) and dengue shock syndrome (DSS) [6]–[7], [4]. In spite of the cross-reactivity observed among the different serotypes, infection with one DENV induces long-term protective immunity only to the same serotype. In fact, several reports suggest that an inappropriate pre-existing immune response against other serotypes results in the increase of severe cases of the disease observed in patients experiencing heterotypic secondary infections [8]–[12].

The DENV genome is constituted of a single positive RNA strand of approximately 11 kb, which is translated into a large polyprotein during virus infection. The polyprotein is processed to yield three structural proteins, capsid (C), premembrane/membrane (prM/M) and envelope (E), as well as seven non-structural proteins, NS1, NS2A, NS2B, NS3, NS4A, NS4B and NS5 [13], [5]. The cleavage of this polyprotein, which represents an essential step for viral replication, is performed by host enzymes and the NS3 viral protease [14]–[16]. The NS3 is a multifunctional protein of approximately 69kDa, involved in the polyprotein processing, RNA capping and RNA replication [5], [16]–[17]. It contains a serine protease domain within the first 180 amino acids at the N-terminal portion, while the 1–167 residues seems to be the minimum sequence required for the proteolytic activity in DENV2 [14], [18]–[19], which is dependent on the association with the co-factor NS2B [20], [15]. The final two-thirds of NS3 protein, in the C-terminal region, contain three other enzymatic domains: RNA-stimulated nucleoside triphosphatase (NTPase), RNA helicase and RNA 5′-triphosphatase (RTPase). The helicase domain unwinds RNA during viral RNA replication and energy for reaction is provided by the NTPase activity [21]–[24]. In addition, the RTPase activity catalyzes the cleavage of the γ-β phosphoric anhydride bond of 5′-triphosphorylated RNA, important for RNA 5′- capping [25].

The development of an effective vaccine against dengue became a priority for the World Health Organization, due to the increasing incidence of severe cases of the disease and its large geographical extension in the world. However, one of the main obstacles for developing such vaccine is the requirement of activating a protective immune response against all four DENV serotypes, without the risk of inducing severe disease [26]–[28]. The dengue infection elicits different immune responses towards the viral proteins. Antibodies are generated mainly against the virus surface E protein and the secreted NS1 protein [29]–[31], while the majority of T-cell epitopes are concentrated within the NS3 protein, the main target for CD4+ and CD8+ T cell response [12], [29], [32]–[33]. Vaccines against flavivirus are generally based on the E protein, which contains most of the epitopes that elicit neutralizing antibodies [26], [32]. However, this protein may also induce non-neutralizing antibodies involved in the phenomenon of antibody-dependent enhancement (ADE) of DENV infection, which can be associated to the occurrence of increased numbers of DHF in secondary infections [8], [34]–[35]. Alternatively, some reports suggest the use of non-structural proteins for dengue vaccines to overcome such problem [36]–[38]. The NS1 is also highly immunogenic and may generate antibodies with complement fixing activity, probably triggering the lyses of infected cells which present this protein on its surface [36]–[37], [39]. Nevertheless, antibodies against the NS1 may also cross-react with human proteins, which can be associated to some pathological effects of the dengue infection [40]–[42]. In contrast, there are only few studies evaluating the use of the NS3 protein as a protective antigen against DENV, as well as other virus from the *Flaviviridae* family. Immunization with the NS3 from flavivirus, in general, induced only marginal protection in different animal models [43]–[45]. Studies with the NS3 from DENV, in its turn, reported that mice inoculated with monoclonal antibodies against this protein showed an increase survival time after virus challenge, although most of animals died in the end of the experiment [46]. Further studies suggested that the combination of NS3 with other flavivirus proteins may have a synergetic effect, leading to the increase of protection rates against virus challenge [47]–[48]. On the other hand, other reports suggested the involvement of an immune response against the NS3 in the pathogenesis of DHF [49]–[51].

Therefore, in the present work we evaluated the potential of the NS3 as a protective antigen against DENV2 using DNA vaccines. Different plasmids were constructed encoding the full-length NS3 of DENV2 or only its functional domains (protease or helicase), fused or not to a signal peptide in order to secret the recombinant protein to extracellular medium. Balb/c mice were immunized with these DNA vaccines and challenged with a lethal dose of DENV2. Inoculation with plasmids encoding the protease domain did not conferred any protection, while most animals immunized with vaccines based on the helicase domain or the full-length NS3 protein survived virus infection, although several of them presented some morbidity after challenge. The induction of a cellular immune response, with the production of INF-γ by CD8+ T cells, was detected in animals inoculated by vaccines containing the entire NS3 gene. As far as we know, this is the first study showing protection generated by DNA vaccines based exclusively on the dengue NS3 protein.

## Materials and Methods

### Virus and cell lines

The dengue 2 virus strain New Guinea C (NGC DENV2) was used for cloning sequences encoding the NS3 protein or its functional domains and for challenge assays. The DENV2 used for RNA extraction and cloning was propagated in cells of *Aedes albopictus* (C6/36) (ATCC, USA) in L15 medium (Sigma, USA), supplemented with 10% fetal bovine serum (FBS, Invitrogen, USA). The DENV2 used in challenge assays was obtained from brains of newborn infected mice and propagated in Vero cells (ATCC) cultivated in medium 199 with Earle salts (E199, Sigma), buffered with sodium bicarbonate and supplemented with 10% FBS. For the detection of *in vitro* expression of recombinant proteins, baby hamster kidney cells (BHK-21) (ATCC) were propagated in Dulbecco's Modified Eagle Medium (DMEM) (Invitrogen, USA), supplemented with 5% FBS.

### Plasmid constructions

Six plasmids based on the pcDNA3 vector (Invitrogen) were constructed encoding the full-length NS3 sequence or its functional domains (protease or helicase). Total RNA from C6/36 cells infected with DENV2 was extracted with Trizol (Invitrogen), according to the manufacturer protocol, and used as template for the synthesis of a cDNA. The reaction was performed by reverse transcriptase M-MLV (Invitrogen) with a forward primer, 5′-GGG GGA TAT CGA TAG TGG TTG CGT TG-3′, which hybridizes to nucleotides 2422–2437 in the NGC DENV2 sequence (GenBank M29095). Such cDNA was then used as template for amplification of different NS3 segments by PCR using forward and reverse primers (Table 1), containing *Eco*RV and *Xba*I restriction sites, respectively. The PCR was performed as follows: 2 min at 94°C, followed by 30 cycles of 1 min at 92°C, 1 min at 55°C and 2 min at 72°C, with an extension step of 5 min at 72°C. The different amplified products were electrophoresed on a 1% agarose gel, recovered with glass beads, geneclean (Bio 101, USA), restricted with *Eco*RV and *Xba*I, and ligated to plasmids previously digested with these same enzymes. The pcDNA3 vector (Invitrogen) was used for construction of pcNS3, pcNS3H and pcNS3P plasmids, while the pcTPANS3, pcTPANS3H and pcTPANS3P plasmids were constructed based on the pcTPA, a modified pcDNA3 vector that contains the human tissue plasminogen activator (t-PA) signal sequence [52]. The *Escherichia coli*, strain DH5α, was transformed with recombinant plasmids, which were screened by restriction mapping and confirmed by sequencing (ABI PRISM dye terminator, Applied Biosystems, USA, performed by the Genomic Plataform DNA Sequencing, PDTS-Fiocruz). Plasmids were further isolated and purified by Qiagen Plasmid Giga Kit (Qiagen, Germany), according to manufacturer's instruction. DNA concentrations were quantified by optical density at 260 nm and the integrity of plasmids was checked by agarose gel electrophoresis. Plasmids were suspended in sterile water and stored at −20°C until use.

**Table 1:** Primers used for amplifications of different DENV2 NS3 sequences for construction of the different recombinant plasmids.

vector^a^	primers	Amplified region^b^	Recombinant plasmid
pcTPA	5′-GGGGGATATCGCTGGAGTATTGTGGG-3′^c^	protease (4522–5076)	pcTPANS3P
	5′-GGGTCTAGA **TTA**CTTTCGAAAAATGTCATC-3′^d^		
	5′-GGGGATATCAGAAAATTGACCATCATGG-3′^c^	helicase (5077–6375)	pcTPANS3H
	5′-GGGGTCTAGA **TTA**CTTTCTTCCAGCTGCA-3′^d^		
	5′-GGGGGATATCGCTGGAGTATTGTGGG-3′^c^	NS3 (4522–6375)	pcTPANS3
	5′-GGGGTCTAGA **TTA**CTTTCTTCCAGCTGCA-3′^d^		
pcDNA3	5′-GGGGGATATC **ATG**GCTGGAGTATTGTGGG-3′^c^	protease (4522–5076)	pcNS3P
	5′-GGGTCTAGA **TTA**CTTTCGAAAAATGTCATC-3′^d^		
	5′-GGGGATATC **ATG**AGAAAATTGACCATCATGG-3′^c^	helicase (5077–6375)	pcNS3H
	5′-GGGGTCTAGA **TTA**CTTTCTTCCAGCTGCA-3′^d^		
	5′-GGGGGATATC **ATG**GCTGGAGTATTGTGGG-3′^c^	NS3 (4522–6375)	pcNS3
	5′-GGGGTCTAGA **TTA**CTTTCTTCCAGCTGCA-3′^d^		

a Vectors used for cloning.

b Genome coordinates [64].

c Forward primer (start codon is marked in bold type and *Eco*RV restriction site is underlined).

d Reverse primer (stop codon is marked in bold type and *Xba* I restriction site is underlined).

### Transfection of BHK cells with different plasmids

BHK cells were transiently transfected with recombinant plasmids or control vectors (pcDNA3 or pcTPA) and fixed or harvested 24 h after transfections. For detection of recombinant proteins by immunofluorescence, 2×10^4^ cells/well were plated in chamber slides (Nunc, Denmark) with Optimen medium (Invitrogen) and transfected with 0.2 µg of each DNA, while for western blot, 5×10^5^ cells were plated in 25 cm^2^ bottle and transfected with 2 µg of each DNA. Transfections were performed 24 h after cell plating, using lipofectamine (Invitrogen) under conditions suggested by the manufacturer.

### Immunofluorescence

Monolayers of transfected cells were washed with 0.1 M phosphate buffer (PB), pH 7.4, fixed with 4% paraformaldehyde for 10 min, permeabilized with 0.6% saponin for 10 min and blocked with 1% bovine serum albumin (BSA) and 0.2% saponin for 15 min. Cells were then incubated for 1 h at 37°C with a mouse polyclonal antibody against DENV2 NS3 protein that was expressed in *E. coli*, washed tree times with PB and incubated for 1 h at 37°C with fluorescein-conjugated goat anti-mouse IgG (Southern Biotechnology, USA), diluted 1∶100 in PB. After washing again with PB, slides were mounted with Vectashield medium (Vector Laboratories Inc., USA) and cells were visualized in a fluorescence microscope (Nikon Eclipse E600).

### Western blotting

Western blotting of transfected whole-cell extracts and culture supernatants was performed as previously described [38]. Proteins present in culture supernatants were precipitated by adding 100% trichloroacetic acid (TCA) to a final concentration of 10% (V/V) and pellets were washed twice with ice-cooled 70% acetone. Cell extracts and TCA precipitated proteins were suspended in SDS-PAGE sample buffer [53] and boiled for 5 min. Proteins were sorted in SDS-PAGE (12.5% total acrylamide concentration) and transferred into nitrocellulose membranes. Recombinant proteins were detected with mouse polyclonal antibodies against the DENV2 NS3 protein, followed by incubation with rabbit anti-mouse IgG conjugated to horseradish peroxidase (Southern Biotechnology), diluted 1∶4000. Membranes were developed with the ECL kit (Amersham Biosciences, UK) and exposed to Kodak X Omat films.

### Mouse immunization and virus challenge

Experiments with mice were conducted in compliance with ethical principles in animal experimentation stated in the Brazilian College of Animal Experimentation and approved by the Animal Use Ethical Committee of Fundação Oswaldo Cruz (FIOCRUZ) (approval ID: L-067/08).

Male Balb/c mice, 4 to 6 weeks old, were inoculated by the intramuscular (i.m.) route with 50 µg of plasmid dissolved in 50 µL of phosphate buffer saline (PBS) in each tibialis posterior muscles (100 µg/mouse) using 27-gauge needles. Each animal group (n = 10 or n = 20) received two doses of one recombinant plasmid (pcTPANS3, pcTPANS3H, pcTPANS3P, pcNS3, pcNS3H or pcNS3P) or control vectors (pcTPA or pcDNA3), given 2 weeks apart. Two weeks after the second DNA dose, vaccinated and non-immunized animals were challenged with the mouse brain adapted NGC DENV2. For challenge assays, animals were anesthetized with a mixture of ketamine-xylazine [54] and then inoculated by the intracerebral route (i.c.) with 30 µL of NGC DENV2 corresponding to approximately 40 LD_50_. Mice were monitored for 21 days after challenge, recording mortality and morbidity, expressed mainly by the appearance of hind leg paralysis and alterations in spinal column. Evaluation of different morbidity degrees in each animal group was performed using a scale ranging from 0 to 3 (0 =  none, 1 =  mild paralyses in one hind leg or alteration of the spinal column with a small hump, 2 =  severe paralyses in one hind leg and alteration of the spinal column with a small hump or severe paralyses in both hind legs, 3 =  severe paralyses in both hind legs and deformed spinal column or death). After 21 days, animals that survived challenge were sacrificed and blood samples were collected by cardiac puncture. The protective efficacy of the DNA immunizations was tested in two or one independent challenge assays, for inoculations with vaccines encoding the t-PA signal peptide (pcTPANS3, pcTPANS3H and pcTPANS3P) or injections with plasmids without this sequence (pcNS3, pcNS3H and pcNS3P), respectively.

### Interferon-γ ELISPOT assay

Spleen cells from mice inoculated with the pcTPANS3 or pcNS3 vaccines, as well as with the control vectors pcDNA3 and pcTPA, were used in IFN-γ ELISPOT test. The assay was performed with a 9-mer peptide (GYISTRVEM), corresponding to 298–306 amino acid residues of the DENV2 NS3 protein, which is an immunodominant T cell epitope in Balb/c mice [55]. Groups of animals (n = 6) were immunized with the DNA plasmids, as described above, and splenocytes were collected 4 weeks after the first immunization.

The IFN-γ ELISPOT mouse set (BD Biosciences) was used in accordance with the manufacturer's instruction. Briefly, 96-well filtration plates were coated overnight at 4°C with 5 µg/mL IFN-γ capture monoclonal antiboby (100 µL, in PBS), followed by washing and blocking with RPMI-1640 medium (Sigma), containing 1% L-glutamine and 10% FBS, for 2 hours at room temperature. Red blood lysed splenocytes (10^6^ cells/well) were added in 100 µL RPMI-1640 with the subsequent addition of 100 µL of the NS3 peptide (final concentration of 10 µg/mL). Non-stimulated and concanavalin A (Con A, 5 µg/mL) stimulated cells were used as negative and positive controls, respectively. Splenocytes were cultured for 20 h at 37°C under 5% CO_2_. Plates were washed once with water and then with 0.05% Tween 20-PBS (PBST), followed by incubation for 2 h at room temperature with 2 µg/mL of biotinylated IFN-γ detection antibody, diluted in 100 µL PBS with 10% FBS. Plates were washed again with PBST and incubated for 1 h at room temperature with streptavidin-horseradish peroxidase diluted 1∶100. Spots were revealed with AEC substrate reagent set (BD Bioscience) at room temperature and counted with an Immunospot reader (Cellular Technology Ltd, Cleveland,OH) using the Immunospot Software Version 3. Results were expressed as the average of spot-forming cells number (SFC) per 10^6^ cells, from triplicate wells, after subtraction of background values detected in splenocytes incubated only with medium.

### Statistical analysis

Statistical experimental analyses were performed using the GraphPad Prism software (La Jolla, USA), version 5.02. For the analysis of survival rates, statistical significances were evaluated by chi-square test, while differences in the degree of morbidity and in SFC values were analyzed by Mann-Whitney test. Values were considered significant at P<0.05.

## Results

### Construction of different DNA vaccines based on the NS3 protein or its functional domains

**Figure 1:**
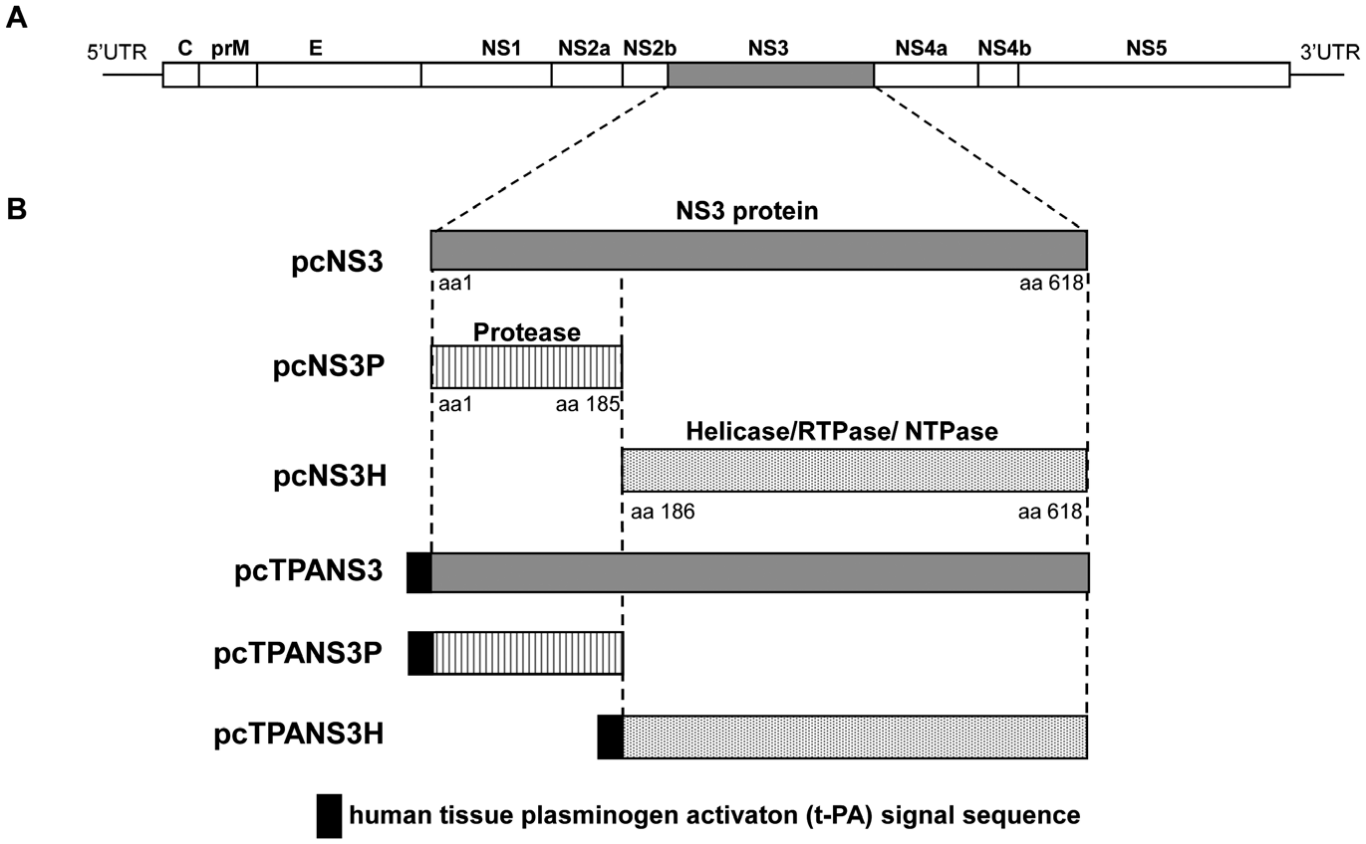
Schematic representation of the DENV2 genome (a) and the regions coded by the different recombinant plasmids (b): pcTPANS3, pcTPANS3P, pcTPANS3H, pcNS3, pcNS3P, pcNS3H. Boxes represent the NS3 region (gray areas), the protease domain (streaked areas) and the helicase/RTPase/NTPase (dotted areas) domain; black box symbolizes the signal peptide derived from the human tissue plasminogen activator (t-PA).

Six DNA vaccines, pcNS3, pcNS3H, pcNS3P, pcTPANS3, pcTPANS3H and pcTPANS3P, were constructed as described in materials and methods and represented in figure 1. The pcNS3 and pcTPANS3 vaccines encode the full-length NS3 protein. The pcNS3P and pcTPANS3P plasmids contain the sequence that codes the first 185 NS3 N-terminal amino acids, which enclose the protease domain, while constructs pcNS3H and pcTPANS3H encode the 433 NS3 C-terminal amino acids (from residues 186 to 618), which correspond to the RTPase/NTPase/helicase domains. Fragments were cloned without any signal sequence (pcNS3P, pcNS3H and pcNS3), for intracellular expression, or in frame with the t-PA signal peptide (pcTPANS3P, pcTPAND3H and pcTPANS3), for the secretion of recombinant proteins (Fig. 1).

### In vitro expression of recombinant proteins

**Figure 2:**
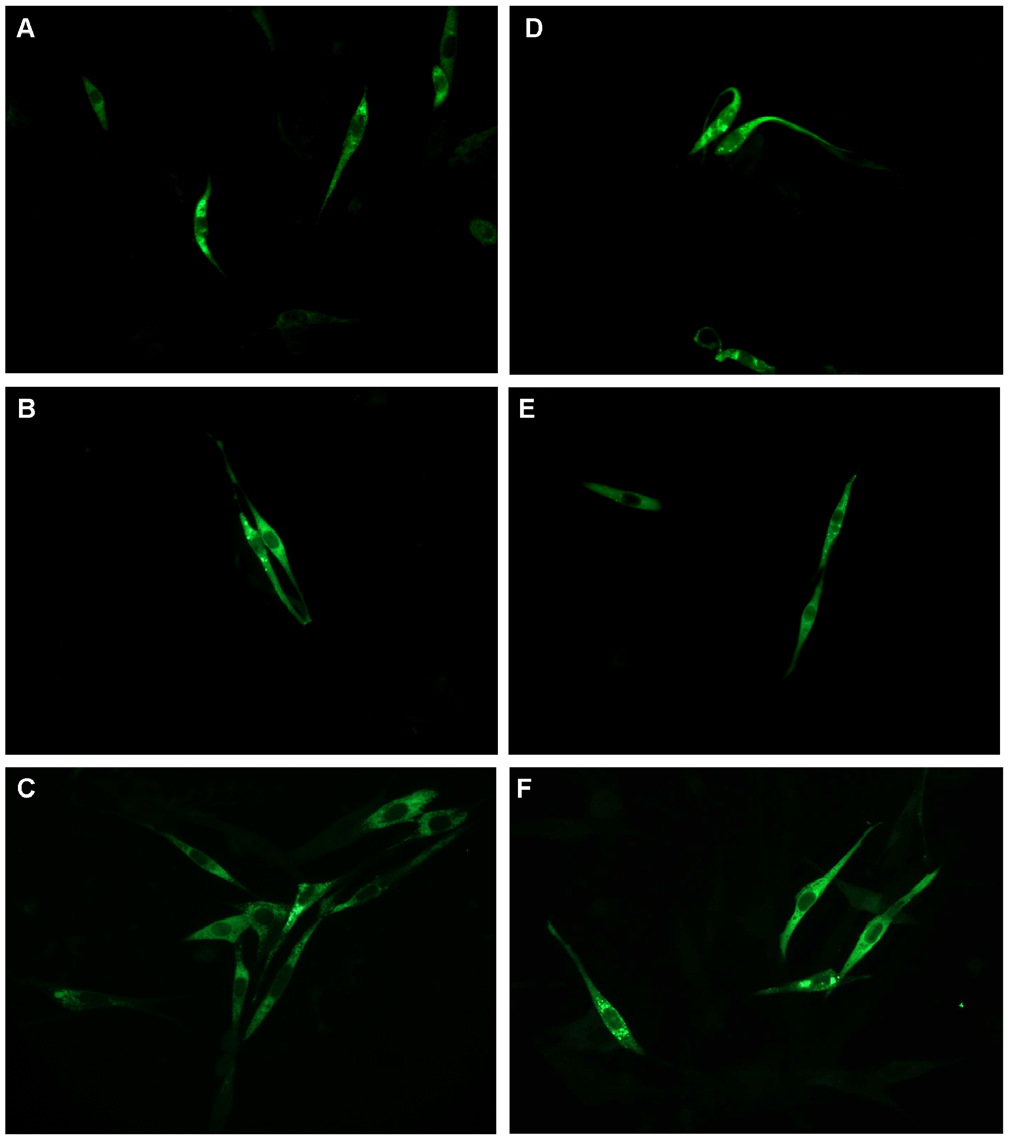
Immunofluorescence of BHK-21 cells transfected with recombinant plasmids based on NS3 protein or its functional domains: pcTPANS3 (a), pcTPANS3H (b), pcTPANS3P (c), pcNS3 (d), pcNS3H (e) and pcNS3P (e). Cells were treated with a mouse polyclonal antibody against the DENV2 NS3 protein. Magnification 400x.

**Figure 3:**
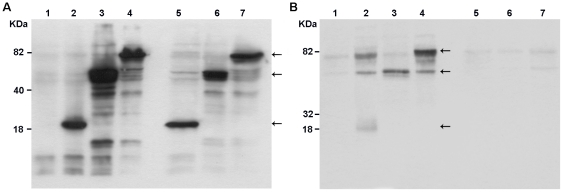
Western blots of whole-cell extract (a) and culture supernatants (b) of BHK-21 cells transfected with the different DNA vaccines. The recombinant proteins were detected in SDS-PAGE, using a mouse polyclonal antibody against the DENV2 NS3, harvested from transfections with pcTPA (lane 1), pcTPANS3P (lane 2), pcTPANS3H (lane 3), pcTPANS3 (lane 4), pcNS3P (lane 5), pcNS3H (lane 6) and pcNS3 (lane 7). Arrows indicate bands corresponding to the protease, helicase and full-length NS3 recombinant proteins.

The expression of recombinant proteins was evaluated in BHK-21 cells transiently transfected with the different plasmids. Immunofluorescence assays revealed positive reaction in cells transfected with all recombinant plasmids, indicating that constructs were able to promote expression of the full-length NS3 protein or its functional domains in eukaryotic systems, regardless the presence of the t-PA signal peptide (Fig. 2). Cells transfected with control vectors (pcTPA or pcDNA3) did not react with NS3-specific antibodies (data not shown). Western blot analysis of transfected cell extracts confirmed the identity of the recombinant proteins, showing predictable molecular weights. A protein of approximately 20 kDa, corresponding to the NS3 protease domain, was detected in pcTPANS3P- and pcNS3P-transfected cell lysates, while extracts from cells transfected with pcTPANS3H and pcNS3H revealed a band around 50 kDa, according to the 433 amino acids of the helicase domain (Fig. 3a). Furthermore, a band of approximately 70 kDa was observed in pcTPANS3- and pcNS3-transfected cell extracts, which corresponds to the full-length NS3 protein (Fig. 3a). Other specific bands, with different molecular weights, were also observed in the extract of cells transfected with plasmids encoding the full-length NS3 or the helicase domain, probably resulting from spontaneous cleavage and/or degradation of the NS3 protein. As expected, NS3-specific recombinant proteins were detected only in the supernatants of cells transfected with plasmids containing the t-PA signal sequence (pcTPANS3P, pcTPANS3H and pcTPANS3), which demonstrated that this signal peptide indeed targeted the proteins to secretion (Fig. 3b). No NS3-specific band was observed in extracts or supernatants of cells transfected with control vectors (Fig. 3).

### Protection generated by the different DNA vaccines encoding NS3 regions

**Figure 4:**
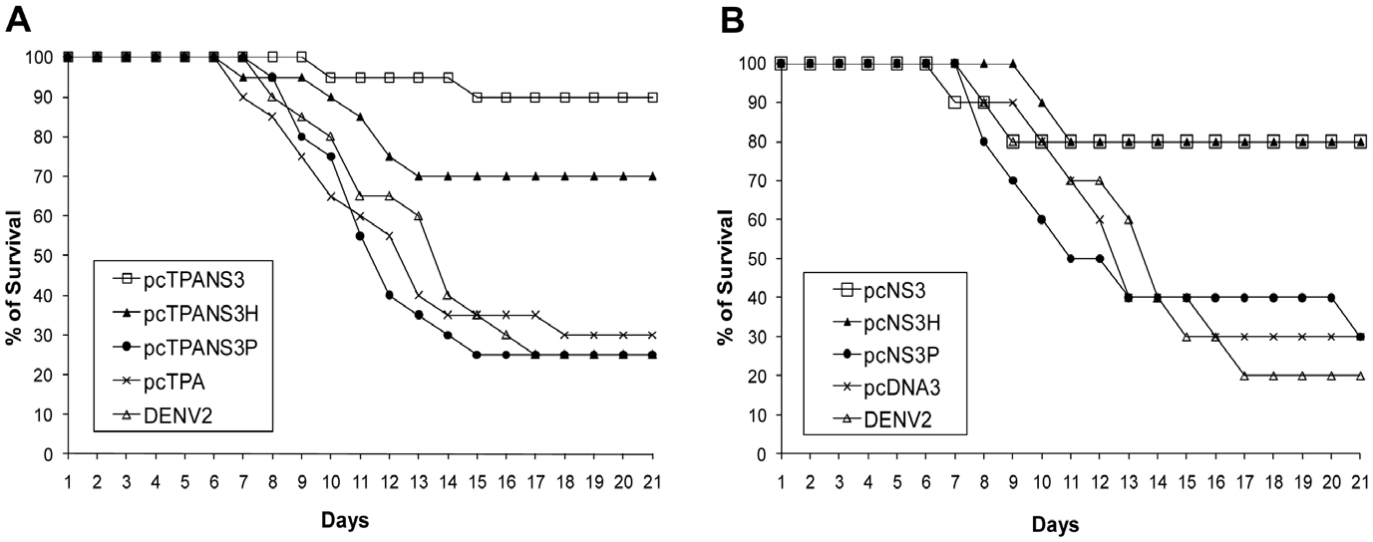
Survival percentage of Balb/c mice immunized with pcTPANS3, pcTPANS3H and pcTPANS3P (a) or pcNS3, pcNS3H and pcNS3P (b) and challenged with the NGC DENV2. Mice were immunized with two DNA doses and challenged 4 weeks after the first plasmid inoculation. Non-immunized (DENV2) and pcTPA or pcDNA3-injected mice followed the same dengue virus infection procedure. Mice were daily monitored and death was recorded. Data represent compilation of two (a) or one (b) independent experiments, with groups of 10 animals in each test (n = 20 and n = 10, respectively).

The protective efficacy of plasmids encoding the full-length NS3 protein or its functional domains was evaluated in mice immunized with such DNA vaccines and challenged with a mouse brain-adapted DENV2. Results indicated that the tested vaccines induced different levels of protection depending on the codified NS3 region, regardless the presence or not of the signal peptide. Animals vaccinated with pcTPANS3 presented 90% of survival rate, while 70% of pcTPANS3H-immunized mice survived virus challenge, although this difference was not statistically significant (Fig. 4a). However, both group showed statistically significant differences of survival rates when compared to control groups, in which only 25% and 30% of non-immunized and pcTPA-inoculated animals, respectively, survived virus inoculation (p<0.0001) (Fig. 4a). Similar results were observed in the mouse groups inoculated with pcNS3 and pcNS3H plasmids, both presenting survival rates of 80%, which was statistically different (p<0.0001) from control animals (20% and 30%, in non-immunized and pcDNA3-inoculated mice, respectively) (Fig. 4b). Furthermore, the presence of the t-PA signal sequence in the constructions encoding either the entire NS3 or the helicase domain did not induce statistical differences in survival rates, when compared to results observed with similar plasmids without this signal peptide. In contrast, none of the DNA vaccines encoding only the NS3 protease domain induced protection against the DENV2 challenge, with survival rates of 25% and 30% for pcTPANS3P- and pcNS3P-immunized mice, respectively, which was similar to those observed in control groups (Figs. 4a and 4b).

**Figure 5:**
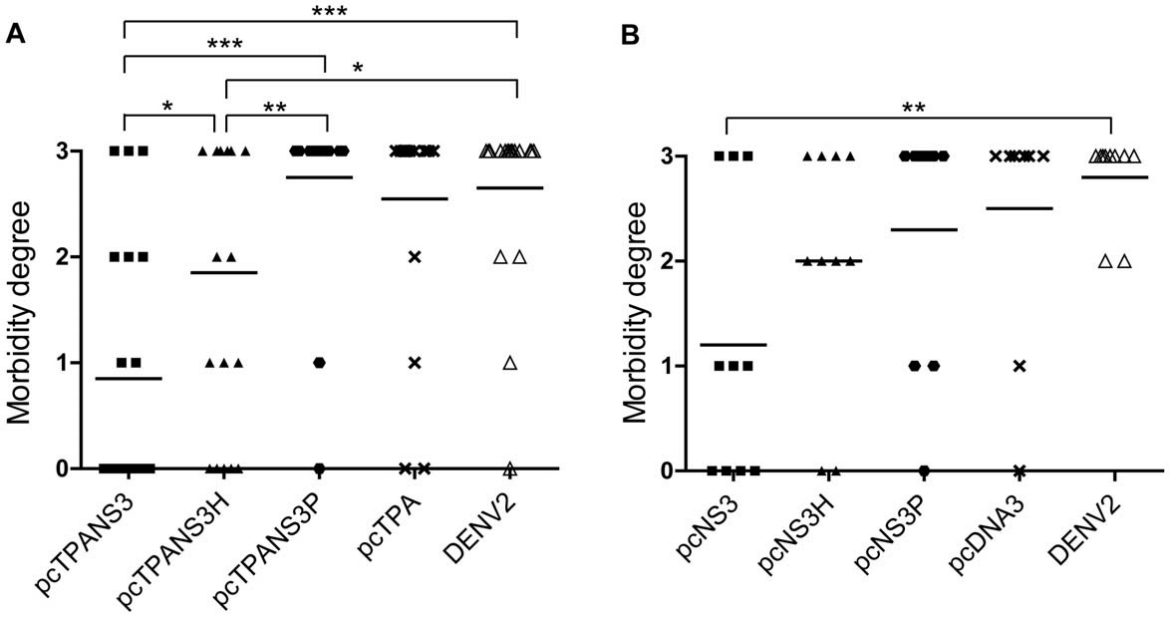
Morbidity degree of Balb/c mice inoculated with pcTPANS3, pcTPANS3H, pcTPANS3P and pcTPA (a) or pcNS3, pcNS3H, pcNS3P and pcDNA3 (b) and challenged with the NGC DENV2. Control groups of non-immunized animals (DENV2) were also challenge with the dengue virus. Clinical signs of infection, mainly hind leg paralysis, alterations in spinal column and deaths, were monitored during 21 days post challenge. The semi-quantitative analysis of morbidity degrees after virus challenge were performed using a subjective scale ranging from 0 to 3 (0 =  none, 1 =  mild paralyses in one hind leg or alteration of the spinal column with small bump, 2 =  one severe hind leg paralyses and alteration of the spinal column with small bump or two severe hind leg paralyses, 3 =  two severe hind leg paralyses and bump deformed spinal column or death). Asterisks indicate statistically significant differences (***, p<0.0001; **, p<0.01; *, p<0.04).

Although most of animals immunized with the DNA vaccines based in full-length NS3 survived virus challenge, 40% and 60% of pcTPANS3- and pcNS3-immunized mice, respectively, showed clinical signs of infection (Fig. 5). In fact, 30% of these animals manifested severe neurological signs (grades 2 and 3). However, the morbidity rate in these groups were significant different comparing to non-immunized mice, in which 95% to 100% of animals presented several clinical signs of infection (p<0,0001 for pcTPANS3 and p<0,01 for pcNS3) (Figs. 5a and 5b). In contrast, 75% to 80% of mice inoculated with the vaccines containing the helicase domain and 90% of animals injected with plasmids based on the protease sequence presented clinical signs after challenge (Fig. 5), and these morbidity degrees were not statistically different from that observed in non-immunized groups. Furthermore, results showed that pcTPANS3H-immunized animals presented statistically higher morbidity degrees when compared to mice vaccinated with the pcTPANS3 (p<0.04). Thus, data indicated that only immunization with plasmids based on the full-length NS3 protein confered some protection, when mortality and morbidity parameters were taken together.

### Production of IFN-γ induced by DNA vaccines encoding the NS3 region

**Figure 6:**
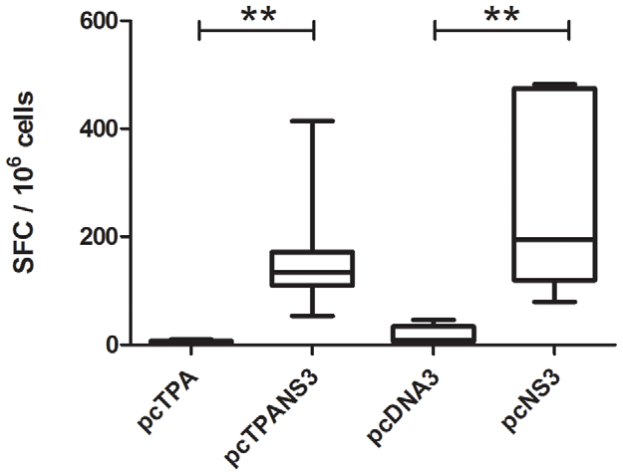
Production of INF-γ by T cells of animals immunized with the DNA vaccines encoding the full-length NS3 protein. Splenocytes collected from groups of mice (n = 6) inoculated with pcTPANS3, pcNS3, pcDNA3 or pcTPA plasmids were stimulated with a T-cell specific peptide and the number of spot-forming cells (SFC) were quantified in a 24h ELISPOT assay. Asterisks indicate statistically significant differences (**, p<0.003).

An IFN-γ ELISPOT assay was performed in order to investigate the T-cell response induced with plasmids encoding the full-length NS3 protein, using a previously identified NS3 immunodominant epitope in Balb/c mice. All animals immunized with either the pcTPANS3 or pcNS3 DNA vaccines presented higher and statistically different SFC values when compared to the control groups, inoculated with pcDNA3 or pcTPA plasmids (Fig. 6). Overall, animals vaccinated with the pcNS3 presented higher frequencies of NS3-specific IFN-γ-secreting cells comparing to pcTPANS3-immunized mice, although such differences were not statistically significant (Fig. 6). Positive control using Con A as stimulating antigen confirmed the cell viability (data not shown).

## Discussion

The NS3 is a conserved protein among the different dengue serotypes, which elicits a strong cellular immune response after viral infection in humans and animal models [5], [12], [29], [32]. Although there are several studies analyzing such immune responses, little is known about the ability of this protein to induce protection. Therefore, in the present work, we tested different DNA vaccines either encoding the full-length NS3 protein or only its functional domains, protease and helicase, in order to evaluate their protective potential in mice.

The DNA vaccines were constructed with or without a signal peptide for secretion of the antigens. All constructs were able to mediate the expression of recombinant proteins *in vitro*, evaluated in BHK-21 cells transfected with the different plasmids. However, secretion of NS3, as well as its protease and helicase domains, was only achieved in cells transfected with plasmids containing the t-PA signal sequence, thus confirming that this peptide was efficient for targeting translocation of such proteins into the endoplasmic reticulum and golgi complex. Other reports with different genes, including those from our group, also used the t-PA signal peptide in DNA vaccines, in order to mediate the secretion of recombinant antigens. In these cases, protein secretion seemed to be essential for the induction of a robust immune response, particularly regarding the production of antibodies [38], [56]–[58]. Nevertheless, in the present work, after immunization with plasmids encoding the t-PA signal sequence, we observed only a weak antibody response against the NS3, detected in 1/5, 2/5 and 3/5 animals inoculated with pcTPANS3H, pcTPANS3P and pcTPANS3 DNA vaccines, respectively, with titers ranging from 200 to 1500 (data not shown). Such response were of a low magnitude, especially when compared to DNA vaccines encoding other dengue virus antigens fused to the t-PA peptide, in which specific antibody titers reached approximately 30,000 [38], [52]. Actually, the NS3 protein seems to be a weaker inducer of antibody responses after dengue infection [29], [30]. On the other hand, a cellular immune response with the production of INF-γ by CD8+ T cells was observed in mice immunized with the DNA vaccines encoding the NS3 protein fused or not with the t-PA sequence. Therefore, secretion of the antigen does not seem to be essential for the induction of such immune response, at least in the case of our constructions.

Protection induced by the DNA vaccines encoding the full-length NS3 protein or its functional domains was evaluated in Balb/c mice challenged with a lethal dose of DENV2. Survival rate analyses after viral infection showed that plasmids encoding only the protease domain did not ensure any protection, since results were similar to those observed in control groups (non-immunized or pcTPA- and pcDNA3-inoculated animals). In fact, all the challenge experiments were performed in adult mice, due to the immunization protocol, and animals at this age are naturally less susceptible to DENV infection, which may explain the survival rates observed in control groups. However, severe morbidity was observed in most of these animals. In contrast, vaccines based on the NS3 complete sequence or the helicase portion induced high survival rates, regardless of the t-PA signal peptide presence. Nevertheless, such protection was partial when we evaluated morbidity in vaccinated mice after the virus challenge. In this case, several animals presented infection clinical signs, some of them with high degrees of morbidity. However, in general, mice immunized with the vaccines encoding the full-length NS3 protein exhibited less morbidity, concerning either rates or degrees, when compared to the other groups.

Although the protection observed in our work was not complete, as far as we know, this is the first report showing that NS3 can, in fact, induce a protective response against DENV. Young *et al.* [45] described a partial protection elicited in calves immunized with a DNA vaccine encoding the NS3 protein from the bovine viral diarrhea virus (BVDV), another virus from the *Flaviviridae* family. In this study, authors observed that vaccination prevented fever and virus establishment in the nasal mucosa in 40% of these animals. Konishi *et al.* [44], in turn, reported that DNA immunization with a plasmid containing the NS3 gene from the Japanese encephalitis virus (JEV), also a flavivirus, elicited protection in 50% of mice challenged with a relatively low virus dose. However, such results were not reproducible when a high challenge dose was used. Moreover, studies with one other flavivirus, employing a DNA vaccine based on the NS3 from the tick-borne encephalitis virus (TBEV), revealed absence of protection in mice [43]. On the other hand, the combination of vaccines encoding NS3 as well as another virus protein seemed to have a synergic result, increasing protection levels [47]–[48].

Regarding dengue studies, Tan *et al.* [46] stated that passive immunization with monoclonal antibodies against NS3 protracted the mean time of survival in mice challenged with a lethal dose of DENV1. However, although the mechanism involved in the protection elicited by NS3 has not so far been established, the humoral immune response against this protein probably only plays a marginal role since it is a non-structural protein mostly present inside infected cells. Thus, anti-NS3 antibodies would unlikely have access to this protein and, therefore, would not have a neutralization activity during virus infection. Nevertheless, after infection the DENV NS3 elicit antibodies in humans, mainly in secondary infections, although the predominant humoral immune response is directed to the E and NS1 proteins [29]–[31], [59], which seems to contribute to viral clearance [36], [39], [60]–[61].

On the other hand, NS3 is an important target for T cell responses. Studies with DENV infected patients or volunteers receiving candidate vaccines evidenced CD4+ and CD8+ T-cell epitopes throughout the entire extension of this protein [12]. Moreover, an epitope for CD8+ T cells was also identified in H-2K^d^-restricted mice (Balb/c), corresponding to amino acids 298–306 (GYISTRVEM) of DENV2 NS3, which are in the helicase domain [55]. In accordance with such findings, we observed that the DNA vaccines encoding the full-length NS3 induced a cellular immune response against this epitope with the production of INF-γ. Therefore, results suggest the participation of a cellular immune response in the mechanism of protection induced by the DNA vaccines based on the NS3 protein. In fact, protection was only evidenced in animals immunized with the DNA vaccines encoding either the full-length protein or the helicase domain, both presenting this immunodominant T cell epitope. In contrast, no protection was detected in mice inoculated with the DNA vaccines encoding only the protease domain, which does not contain this epitope.

There is little information about the association of specific T cell responses and protection against either primary or secondary DENV infection. Recently, Yauch *et al.* [62] demonstrated an important role for CD8+ T cells in the host defense. On the other hand, activation of memory CD8+ T cells during heterologous secondary DENV infection appears to result in cytokine storms, characteristic of DHF/DSS [11], [50], [63]. Therefore, further studies will be necessary for the establishment of the mechanisms involved in the protection induced by the DNA vaccines based on the NS3 protein. The cellular immune response elicited by these vaccines seems to be important for protection, which probably involves activation of cytotoxic cells that can destroy infected cells, thus preventing virus spread. However, the partial protection observed in challenged animals indicates that such response is not sufficient and other branch of the immune response should also be activated. Hence, we cannot exclude the possibility of combining immunization with other dengue vaccines, encoding different viral proteins, which might synergistically increase protective immune responses.
